# Development of automatic insect-tracking robot system for measuring local activity changes in free walking

**DOI:** 10.3389/frobt.2025.1602867

**Published:** 2025-06-02

**Authors:** Ryoko Sekiwa, Tatsuya Ibuki, Shunsuke Shigaki

**Affiliations:** ^1^ Department of Electronics and Bioinformatics, Meiji University, Kawasaki, Japan; ^2^ Principles of Informatics Research Division, National Institute of Informatics, Tokyo, Japan

**Keywords:** automatic insect-tracking robot system, odor source localization, silk moth, electroantennogram, adaptive-behavior

## Abstract

This study aims to develop a robotic system that autonomously tracks insects during free walking to elucidate the relationship between olfactory sensory stimuli and behavioral changes in insects. The adaptability of organisms is defined by their ability to select appropriate behaviors based on sensory inputs in response to environmental changes, a capacity that insects exhibit through efficient adaptive behaviors despite their limited nervous systems. Consequently, new measurement techniques are needed to investigate the neuroethological processes in insects. Traditional behavioral observations of insects have been conducted using free-walking experiments and treadmill techniques; however, these methods face limitations in accurately measuring sensory stimuli and analyzing the factors contributing to detailed behavioral changes. In this study, a robotic system is employed to track free-walking insects while simultaneously recording electroantennogram (EAG) responses at the location of the antenna of the insect during movement, thus enabling the measurement of the relationship between olfactory reception and behavioral change. In this research, we focus on a male silk moth (Bombyxmori) as the target insect and measure its odor source localization behavior. The system comprises a high-speed camera to estimate the movement direction of the insect, a drive system, and instrumentation amplifiers to measure physiological responses. The robot tracks the insect with an error margin of less than 5 mm, recording the EAG responses associated with the olfactory reception during this process. An analysis of the relationship between EAG responses and behavior revealed that the silk moth exhibits a significant amplitude in its EAG responses during the initial odor source localization stage. This suggests that the moth does not necessarily move toward the strongest odor. Further information-theoretic analysis revealed that the moth might be moving in the direction most likely to lead to odor detection, depending on the timing of its olfactory reception. This approach allows for a more natural measurement of the connection between olfactory sensory stimuli and behavior during odor source localization. The study findings are expected to deepen our understanding of the adaptive behaviors of insects.

## 1 Introduction

Living organisms can survive and thrive by choosing appropriate behaviors in a constantly changing environment. This ability is generally called adaptability. Living organisms support this adaptability by receiving environmental stimuli through their sensory organs, processing stimuli in the brain, and converting them into appropriate movements. The nervous system is responsible for processing sensory stimuli and converting them into movements. The basic structure and function of neurons, which are the elements that make up the nervous system, are common to mammals and insects. However, owing to their size, the nervous system of insects is small, with only approximately 100,000–1 million neurons, which is significantly fewer than those of mammals (approximately 100 billion neurons). Even with this small neural system, they can perceive changes in the environment and their body and respond to them by generating various movements. Considering that the diverse and sophisticated behaviors of these insects are generated by a nervous system comprising only approximately one million neurons, it is undeniable that they have energy-saving and highly efficient signal processing and body control mechanisms. Consequently, several attempts have been made to reconstruct insect adaptability in robotic systems (Pfeifer et al., 2012; Webb et al., 2004).

Careful observation of the behavior of the target insect is necessary to investigate their adaptability. This allowed us to analyze the mechanism of behavioral output modulation in response to changes in the environment or body. The simplest way to observe insect behavior is the so-called “free-walking (or flight) experiment,” in which the insect is placed in an open space and made to perform a desired task [e.g., (Rutkowski et al., 2009)]. Behavioral changes can be continuously observed by placing external cameras above or on the side of the space in which the insect moves. However, measuring the sensory stimuli experienced by freely walking insects has long posed a significant challenge. Although such measurements can be achieved through advanced electrophysiological techniques, simultaneously recording from multiple sensory organs remains technically difficult. In response to this limitation, research utilizing *Drosophila melanogaster*, which is highly amenable to tools such as optogenetics and calcium imaging, has made notable strides in the field. For instance, studies employing systems like “Flyception” have successfully enabled quantitative monitoring of brain activity via calcium imaging in freely walking flies (Grover et al., 2016; Grover et al., 2020). Moreover, pioneering research using genetically modified *Drosophila* that exhibit olfactory behaviors in response to light stimulation has elucidated how flies exploit the spatiotemporal dynamics of odor stimuli (Kadakia et al., 2022). While these investigations have advanced our understanding of how sensory inputs are processed and transformed into behavior during free movement, there remains a critical demand to explore navigational behaviors in non-model insects for which genetic tools are not readily applicable. In the study of insect behavior modeling, acquiring comprehensive datasets that link sensory stimuli to behavioral outputs is of paramount importance. Therefore, a treadmill technology for insects has been developed to address this issue by measuring the relationship between sensory stimuli and behavior. The treadmill technology can be broadly divided into the servosphere (Meyer-Rochow, 1973) and tethered methods (Dahmen, 1980). Both are behavioral measurement devices for walking animals, and a virtually infinite plane can be created by having the target organism walk on a sphere. This makes it possible to measure the relationship between sensory stimuli and movement in animals that move over long distances, even in laboratories with limited space.

The basic configuration of the servo sphere comprises a measurement system for capturing the movement of an animal (insect) and a drive system for controlling the sphere. The servo sphere uses an observation system, such as a camera, to estimate the direction of movement of the organism on the top of the sphere; by controlling the sphere in the opposite direction to the direction of movement, the measurement target is always kept on top of the sphere, and the movement trajectory of the organism is recorded. The target organism can be measured without contact using this method; therefore, the servo sphere has the advantage of obtaining a natural movement trajectory similar to that of a free-walking experiment. A three-degree-of-freedom servo sphere that compensates for the position and heading angle of the insect has also been developed recently. Studies have reported that this method makes it possible to simultaneously measure behavioral and physiological responses (Shigaki et al., 2016). However, this method has the disadvantage of applying a certain amount of inertial force to the insect, which causes unnecessary external forces because the sphere is dynamically controlled at each time step. In response to this, the tethered method, in which a part of the body of an organism is fixed and placed on a sphere floating in the air to measure its behavior, has long been used as a behavior measurement system for walking organisms. Mechanical and electrical noises are generated less than in the servo sphere system because the body of an organism is fixed. This method has been widely used as a tool in neuroethology (Paulk et al., 2015; Guo and Ritzmann, 2013; Shigaki et al., 2019b). In addition, because providing sensory stimuli to organisms at a high resolution is possible, a virtual reality system for insects has been proposed, in which multiple stimulators are installed around the insect and linked to a virtual space (Yamada et al., 2021; Ando et al., 2021). However, body deformation may be alienated by fixing the body of the insect. In addition, the insect moves in a different state than the free-walking state. Moreover, in the case of a virtual reality system for insects, the virtual space is a modeled environment, and because the sensory stimuli are artificially reproduced for insects, the sensory stimuli may differ from natural sensory stimuli. If we could observe the state of sensory stimuli while the insect is walking freely, we could obtain a correlation between sensory stimuli and behavioral output in a more natural state, which would contribute to a further understanding of the adaptive behavior of insects.

**FIGURE 1:**
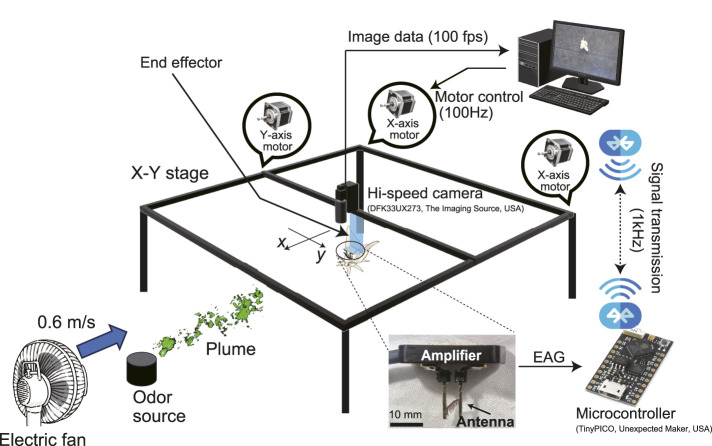
Outline of the proposed automatic insect-tracking robot system.

Therefore, this study primarily aims to develop a robot system that automatically tracks a freely walking insect. This allows us to simultaneously measure local insect activity while measuring behavior to achieve a global goal. The basis of this robotic system is a large X-Y stage, whose end effector is equipped with a high-speed camera to observe the insect. The end effector refers to the moving part of the X-Y stage shown in Figure 1. This high-speed camera is used to estimate the moving direction of the insect, and the end effector can be controlled to track the insect. We evaluated the robot system by measuring the actual insect movement as it performs the odor source localization task. Although odor is an invisible substance, it is a signal that has excellent persistence and diffusibility; therefore, it is a sensory stimulus that insects generally utilize for communication and exploration (Renou, 2014). Owing to technical difficulties, very few studies have examined the relationship between odor stimuli and the behavior of migrating insects in open spaces (Vickers and Baker, 1994). In a previous study, an antenna cut from another insect was attached to an insect scheduled for use in an open-space experiment, and the odor-reception information at the position where the insect moved was measured by recording the potential response from the antenna. The technique of measuring electrical potential responses from the antenna is called an electroantennogram (EAG), which records the sum of the physiological responses of the olfactory receptor cells in the antenna to an odor stimulus. Although measuring the physiological antennal responses of a moving insect is technically difficult, the measured data are highly valuable because they clearly show which odors cause the insect to switch its behavior. Therefore, we employed the insect-tracking robot system proposed in this study to measure the relationship between odor sensory stimuli and the localization behavior of walking insects and analyzed this relationship.

## 2 Problem statement

We aimed to construct a robot system that automatically tracks a freely walking insect and to clarify the relationship between odor reception and behavior. In this study, the target insect was a male adult silk moth (Bombyxmori) , which elicits female localization behavior in response to the sex pheromone (Bombykol) (Kanzaki et al., 1992). The silk moth moves at an average speed of 15 mm/s to reach the female (Yamada et al., 2021). The novel robot system that tracks the silk moth during female localization comprises the following: (1) An observation system and a drive and control system that can adequately track the movement of the silk moth. (2) A measurement system that can reduce electrical and mechanical noise and stably measure and record electrophysiological signals.

The components of the robot system are necessary for the following reasons: The main role of the observation system is to estimate the direction of insect movement, and tracking performance is ensured using a high-speed camera to reduce the amount of insect movement per frame. Moreover, the driving and control system controls the X- and Y-axis motors based on results from the observation system to maintain the position of the end effector of the robot system on top of the insect. The electrophysiological response measurement system is equipped with an instrumentation amplifier attached to the moving end effector, which measures stable electrophysiological responses on the end effector.

In this study, we conducted evaluation experiments to simultaneously measure the EAG and behavior of an insect while it searches for an odor source. Because we needed to measure the EAG response at the position of the silk moth, it was necessary to constantly move the end effector above the silk moth and measure the EAG at that time. Since the body length of a silk moth is approximately 30 mm, we aimed to maintain a positional error of 5 mm or less. By maintaining this error range, we can determine the timing and amount of odor received by the silk moth. Therefore, we designed each element of the robot system to achieve this control objective. Moreover, we measured the relationship between the odor source search behavior and EAG of the silk moth and evaluated the robot system.

## 3 Materials and methods

### 3.1 Study insect

The silk moths used in the experiments were purchased in the pupal stage (Kinshu × Showa; Ehine Sansyu Co. Ltd., Ehime, Japan). Emerged individuals were kept in an incubator at 16° C and allowed to acclimate to the laboratory room temperature (24- 26° C) for at least 15 min before the experiment. Silk moths used for behavioral experiments and EAG recording were 2–5 days old after eclosion. When attaching markers to the silk moth, an adhesive (G17, KONISHI Co.,Ltd., Osaka, Japan) that does not affect behavior was used (Shigaki et al., 2016).

### 3.2 Automatic insect-tracking robot system

The robot system was built based on a commercially available laser processing machine (FABOOL Laser Mini 150 cm × 150 cm, SMART DIYs, Yamanashi, Japan). Details will be given in the following section, and the robot system is equipped with a high-speed camera (DFK33UX273, The Imaging Source, Bremen, Germany), an amplifier (ISD2PAD, Oisaka Electronic Equipment Ltd., Japan) for measuring physiological responses, and a small microcomputer for log transfer. Operations from acquiring camera images to controlling the motor of the laser processing machine are carried out at 100 Hz. The fixtures for attaching the camera and amplifier to the robot system were created using a 3D printer (ABS; Creator3 Pro, Flashforge, Zhejiang, China) and laser processing machine (Etcher Laser Pro, SMART DIYs, Yamanashi, Japan).

### 3.3 Amplifier for EAG recording

It must be recorded through an appropriate amplifier because EAG is a small electrical signal. Based on previous research, the EAG amplifier is equipped with high-pass (0.1 Hz) and low-pass (300 Hz) filters and has an amplification rate of approximately 1,000 times (Shigaki et al., 2024). Electrodes (L-shaped pin headers, Useconn Electronics Ltd., Zhejiang, China) for attaching an antenna are attached to the amplifier, and conductive gel (Spectra360, Parker Laboratories, United States) is used to promote the connection between the antenna and the electrodes. EAG was recorded at a sampling rate of 1 kHz. The acquired EAG data was passed through a software low-pass filter with a cutoff frequency of 10 Hz.

### 3.4 Data analysis

R software (R Core Team) was used to create the histograms. In addition, video footage was analyzed using DeepLabCut (DLC) (Mathis et al., 2018). After analysis with DLC, trajectories and EAG data were integrated using a self-coded MATLAB code (2023b, MathWorks, MA, United States). Information entropy was calculated using Python with a self-coded program.

## 4 Construction of the automatic insect-tracking robot system

Figure 1 shows a conceptual diagram of an insect automatic tracking robot system. The end effector of the robot system was equipped with a high-speed camera to estimate the movement direction of the insect and an instrumentation amplifier to measure electrophysiological responses. The robot system comprises an observation system, a driving and control system, and an electrophysiological response measurement system. Each system is explained in detail below.

### 4.1 Observation system

The proposed system used a high-speed camera (DFK33UX273, The Imaging Source, Bremen, Germany) to measure the moving direction and speed of the insect and tracked the movement of the silk moth without contact. Images were acquired at 100 fps with a resolution of 1280×720. The acquired images were processed using the open-source library OpenCV (ver. 3.4.7, Intel, CA, United States). The maximum translational and angular velocities of the silk moth have been reported to be 32.8 mm/s and 1.0 rad/s, respectively (Takashima, 2010). By using a high-speed camera with 100 fps (Hz) to keep the translational error below 5 mm, the expected error is 0.33 mm/frame, suggesting that the control objective can be theoretically achieved. Therefore, we expected that visual feedback could be used to track the insect sufficiently.

However, to set the control cycle to 100 Hz, the process from measurement to calculation of the control command must be completed within 10 m. Consequently, we need to minimize the load on the image processing system. As in a previous study (Shigaki et al., 2016), we attached two-color markers with diameters of 2 mm to the silk moth and implemented an algorithm to calculate the moving speed and direction of the insect using simple color extraction. In particular, the color of the markers was extracted in the HSV color space, and the center of gravity of each marker was calculated as the marker coordinate. The red marker placed near the head was defined as the position of the moth in the observation coordinate system, and the approximate line connecting the two markers was defined as the heading angle of the moth. The markers were treated as points after image processing. As demonstrated in a previous study (Shigaki et al., 2016), we confirmed that attaching these markers did not interfere with the moth movement.

### 4.2 Driving and control system

**FIGURE 2:**
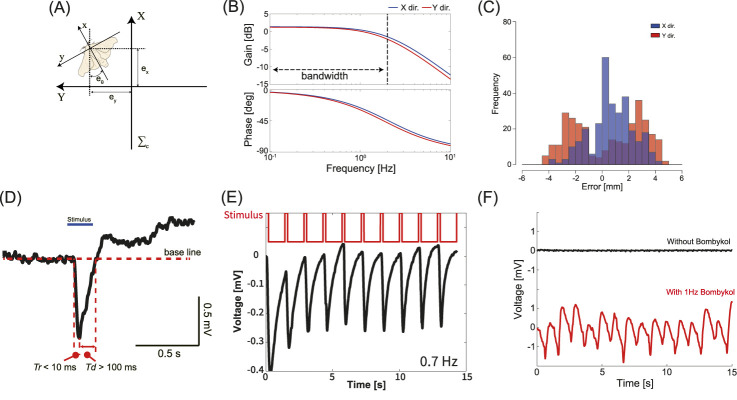
Components of the automatic insect-tracking robot system. **(A)** Definition of the camera coordinate system. Calculating the distance and angle from the camera origin to the silk moth. **(B)** Frequency response characteristics for each axis of the robot system. Both axes are more than twice as high as the silk moth’s movement switching frequency, demonstrating sufficient tracking performance. **(C)** Error when tracking a silk moth performing odor source localization behavior. Both axes are kept to less than 5 mm, allowing the EAG measurement unit to be placed above the silk moth’s head at all times. **(D)** The profile and definition of the EAG response in a static state to a single odor stimulus. $T_{r}$ : Response time, $T_{d}$ : Recovery time. **(E)** Typical EAG response waveform to periodic odor stimulation when the distance between the odor outlet and the antenna is constant. **(F)** Operational confirmation of the physiological response measurement system. The EAG responses are shown when the robot system is subjected to periodic motion, with and without the odor being ejected.

This robot system was based on a laser processing machine (FABOOL Laser Mini 150 cm × 150 cm, SMART DIYs, Yamanashi, Japan). Stepping motors were attached to the X- and Y-axes, and the position of the end effector can be controlled by providing commands to the stepping motors. The position of the end effector can be controlled by transmitting the relative position information in G-code. Relative position was defined as the position error between the origin of the camera coordinate system $\left(\Sigma_{c}\right)$ calculated using the observation system and the insect, as shown in Figure 2A. This position error has the same meaning as the tracking error for evaluating tracking performance.

We employed a proportional-integral-derived (PID) controller to control the robot system. The Ziegler-Nichols’ closed-loop method was used to determine each parameter of the PID control, and the final fine parameter adjustment was performed empirically. The results of calculating the frequency response of the robot system using PID control are shown in Figure 2B. Consequently, the bandwidth was found to be approximately 2 Hz for both the x- and y-axes. The robot system has been suggested to be capable of tracking the silk moth while searching for an odor source because the period during which the silk moth changes its behavior significantly from side to side is approximately 1 Hz or less.

In addition, in a preliminary experiment, we measured the tracking error while the silk moth searched for an odor source. When the tracking error was plotted as a histogram, as shown in Figure 2C, we found that the error while tracking the silk moth was less than 5 mm on both axes, indicating that the control target was achieved.

### 4.3 Physiological response measurement system

An instrumentation amplifier (ISD2PAD, Oisaka Electronic Equipment Ltd., Japan) was attached to the end effector to measure electrophysiological responses. This study aimed to measure the odor reception state during an odor source search using an EAG; therefore, the instrumentation amplifier was customized for EAG measurement. The instrumentation amplifier was equipped with a 0.1 Hz high-pass filter and a 3 kHz low-pass filter and had an amplification rate of approximately 1000 times. The data recorded using this amplifier were collected on a small microcomputer (TinyPICO, Unexpected Maker, Melbourne, VIC, Australia) using an AD converter (MCP3208, Microchip Technology, AZ, United States) and then wirelessly transferred to a recording PC. The sampling rate and EAG recording cycle were set to 1 kHz.

The response profile obtained from EAG measurements in a static condition, where the distance between the odor source and the antennae is fixed, is illustrated in Figure 2D. As depicted in Figure 2D, the time taken to reach the peak following odor stimulation is defined as the response time $\left(T_{r}\right)$ , while the time required for the response to return from the peak to the baseline is defined as the recovery time $\left(T_{d}\right)$ (Shigaki et al., 2019a). In a static context, for single odor stimuli, the response time is typically within 10 milliseconds, and the recovery time generally exceeds 100 milliseconds. However, this is an example measured under static conditions, and these profiles may change if the odor is received in a dynamic situation. In addition, it is important to note that different receptor cells respond to different types of odors; therefore, EAG waveforms differ depending on the odorant. Moreover, in response to periodic odor stimuli, it shows attenuation characteristics as shown in Figure 2E. Figure 2F shows the results of the EAG measurement performed with the robot system turned on and the motor vibrating slightly. An odor stimulus of 1 Hz was emitted from an odor source located approximately 10 cm away. As shown in Figure 2F, the EAG response was measured in response to odor stimulus, demonstrating that the EAG can be measured even in the presence of electrical or mechanical noise.

## 5 EAG-behavioral simultaneous measurement experiment

### 5.1 Preparation for EAG recording

EAG was used to estimate the level of odor reception by silk moths during odor source localization. In this study, the EAG measurements were performed using the antenna of another silk moth, i.e., the “third antenna,” as in the previous study (Vickers and Baker, 1994). The antenna used for the EAG was an adult male silk moth 2–7 days after emergence. The silk moths were purchased in the pupal state (Kinshu × Showa; Ehine Sansyu Co. Ltd., Ehime, Japan). The emerged silkworm moths were managed under a 16:8 h light:dark photoperiod at 18° C and 50–60 % relative humidity using an incubator (CN-40A, Mitsubishi Electric Engineering Co.).

The antenna of a silk moth was cut from its head and attached to the electrodes on an amplifier using a conductive gel (Spectra360, Parker Laboratories, NJ, United States). We confirmed in advance whether the EAG could be correctly recorded from the antenna attached to the electrodes by providing a single shot of the sex pheromone (Bombykol). The antenna was then fixed in the designated position on the end effector. A previous study examining the sensitivity and stability of EAG recording after antennae isolation has shown that the EAG response does not deteriorate within an hour after isolation (Martinez et al., 2014). In the silk moth EAG measurements, preliminary experiments similarly showed that there was no difference in response performance within about 45 min after isolation; therefore, we set the time from antenna isolation to the completion of the simultaneous EAG-behavior experiment to be within 30 min.

### 5.2 Conditions for odor-source search experiment

**FIGURE 3:**
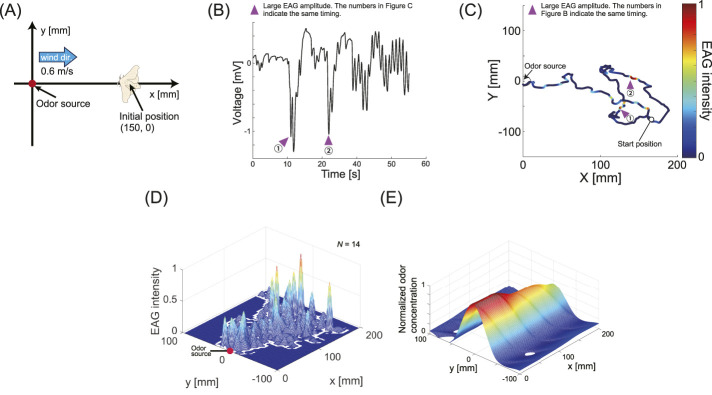
Results of the simultaneous EAG-behavior measurement experiment. **(A)** Field conditions for the localization experiment. The silk moth begins searching from a position 150 mm away from the odor source. **(B)** A typical example of EAG time series data. We confirmed that the amplitude changes over time. **(C)** The relationship between the localization trajectory and EAG intensity. Redder colors indicate larger EAG amplitude, and bluer colors indicate smaller amplitude. The purple triangles in **(B,C)** represent EAG timings with large amplitudes, and their numbers correspond to each other. **(D)** EAG intensity map for all individuals ( N = 14). It is suggestive that EAG intensity is greater at the beginning of searching. **(E)** Odor map of the experimental field measured with an odor sensor.

As illustrated in Figure 3A, odor source localization experiments were conducted in a flat open area. Bombykol, the sex pheromone of a female silk moth, was used as the odor source at the origin of the experimental field ( x , y ) = (0, 0)[m]; it was placed as the odor source. A 1000-ng concentration of bombykol was applied to a filter paper with a diameter of 10 mm. The pheromone-coated filter paper was replaced after each experiment. Air was blown over the filter paper at a flow rate of 4.0 L/min to disperse the odor into the surrounding space. The pheromone concentration employed in this experiment corresponds to the average amount of bombykol emitted by female silk moth (Fujiwara et al., 2014). Furthermore, females have been reported to release bombykol at a frequency of 0.79 ± 0.05 Hz (Fujiwara et al., 2014), and accordingly, in past behavioral experiments, a similar value was used, 1 Hz (duration: 0.2 s, interval: 0.8 s) (Yamada et al., 2021; Kanzaki et al., 1992). Therefore, in this study, the air used to deliver the odor was regulated by an electromagnetic valve, and the odor was emitted at a frequency of 1 Hz (duration: 0.2 s, interval: 0.8 s). A electric fan (YLS-18, Yamazen Corporation, Osaka, Japan) was positioned 0.5 m behind the odor source to facilitate the diffusion of the pheromone. The voltage was controlled so that the wind speed generated by the fan was approximately 0.6 m/s at the odor source. This wind speed was the same as in previous silk moth behavior experiments (Kanzaki et al., 1992). The silk moth was positioned at ( x , y ) = (150, 0)[mm], and the odor-source localization experiment commenced. The initial position from the odor source was also designed based on previous behavioral experiments with silk moths (Kanzaki et al., 1992). Localization was considered successful when the silk moth reached a position within a 10-mm radius of the odor source. However, localization was considered to have failed if the silk moth failed to achieve localization within 180 s. The movements of the silk moth were captured using a camera (FDR-AX45A, Sony, Tokyo, Japan) installed on the side of the field and converted into trajectory data. These experimental conditions were set to closely follow those used in previous experiments with freely walking silk moths (Kanzaki et al., 1992). The silk moths used in the odor source localization experiments were 2–7 days old after emergence. Before starting the behavioral experiment, the silk moths were removed from the incubator, markers were attached, and they were allowed approximately 1 h to become accustomed to the temperature of the behavioral experiment space. Fourteen silk moths were used in the odor source localization experiment.

### 5.3 Results of the odor-source localization experiment

The results of the odor-source search experiment revealed that all 14 individuals successfully localized the odor source (see Supplementary Video S1). The time required for odor-source localization was 24.8 ± 16.2 s. Due to interindividual variation in body size, discrepancies in locomotion speed were observed, resulting in variability in the time required for odor source localization. Nevertheless, it was demonstrated that all silk moths, regardless of differences in body size and associated locomotor speed, were capable of successfully localizing toward the odor source.

**FIGURE 4:**
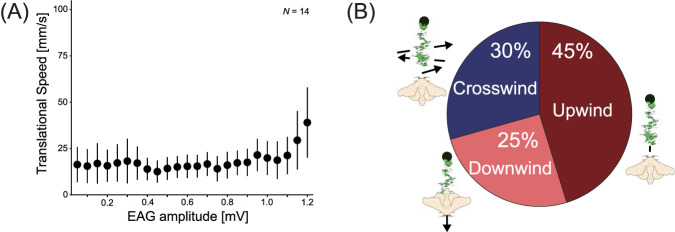
Results of behavioral analysis. **(A)** Relationship between EAG amplitude and translational velocity. **(B)** Proportions of upwind and crosswind movements in individuals exhibiting cast-like behavior.

Figure 3B shows typical time-series data of EAG responses from a simultaneous EAG measurement experiment and behavior during odor-source localization. The time-series data revealed that odor reception began immediately after the experiment began. The EAG amplitude was defined as the EAG intensity, and the localization trajectory was displayed in a color corresponding to the EAG intensity to visualize the relationship between odor reception and behavior. Figure 3C shows the relationship between the EAG intensity and trajectory. The EAG intensity was normalized between 0 and 1; the closer the intensity to 1, the higher the concentration of the odor received. Places with strong EAG intensity are marked with numbers, as shown in Figures 3B,C. We found that strong odor reception resulted in straight-line movement. To quantitatively assess this phenomenon, we plotted the relationship between the EAG amplitude and translational speed, as illustrated in Figure 4A. The results indicate that once the EAG amplitude exceeds 1 mV, the translational speed becomes markedly greater compared to conditions in which the amplitude remains lower. This phenomenon is similar to previous research results that investigated the relationship between odor reception and straight-line behavior using a tethered measurement system (Takasaki et al., 2012). Experiments with this robot system have revealed that the same phenomenon is observed during actual odor-source localization. Furthermore, as shown in Figure 3C, the silk moth approached the odor source by exhibiting cast-like behavior commonly observed in flying insects, with 7 out of 14 individuals demonstrating this phenomenon. To quantitatively show which direction silk moths that exhibited cast-like behavior moved after encountering an odor, we calculated the moving ratio in the upwind, downwind, or crosswind direction as shown in Figure 4B. As a result, we found that while a silk moth moved in the upwind direction about 50 % , it also moved in the downwind and crosswind directions. This is because the behavioral strategy of silk moths differs from that of flying insects, and when they encounter an odor, they adopt a surge behavior in which they move in the encountered direction (Takasaki et al., 2012). However, looking at the global trajectories, it was suggested that they also move in the crosswind direction to gain a chance to encounter an odor.

The relationship between this trajectory and the EAG intensity for all individuals is depicted in Figure 3D as a three-dimensional color map. For comparison, an odor concentration map of the experimental field, generated using an artificial olfactory sensor (MiCS-5524 Gas Sensor Breakout, Adafruit Industries, NY, United States), is presented in Figure 3E. When employing the artificial sensor, it is evident that odor concentration increases in proximity to the odor source. However, from Figure 3D, we can see that for all insects, the EAG intensity tends to be higher during the early stages of searching, away from the odor source. A new insight emerged for comprehensive decision-making based on the time-series data in Figure 3B and the three-dimensional color map in Figure 3D: The amplitude of the EAG did not necessarily increase as the insect approached the odor source, where the odor concentration was expected to be higher. The ratio of the maximum EAG amplitude observed during localization behavior to the EAG amplitude measured closest to the odor source was calculated to be 0.47 ± 0.28 ( N = 14). This indicates that, in proximity to the odor source, the EAG output is reduced to approximately half of its maximal response. This suggests that the silk moth does not simply move toward areas with higher odor concentrations. As one approaches the odor source, exposure to odor stimuli becomes increasingly periodic; however, EAG responses exhibit an attenuation characteristic in response to such periodic stimulation. Figure 2E shows a typical EAG response to 0.7 Hz odor stimulation, with the amplitude decreasing after multiple odor stimulations. This decrease in amplitude recovered to some extent if the antenna did not receive any odor stimulation for approximately 5 s, suggesting that it reduced the response sensitivity to continuous odor stimuli near the odor source. Instead, it effectively reduces the distance to the odor source based on other cues. Next, we evaluated these experimental results using an information-theoretic approach to further analyze the behavior of the insects in greater detail.

### 5.4 Information entropy-based analysis

The factors enabling odor source localization in the silk moth (Bombyxmori) were analyzed from an information theory perspective. In particular, a quantitative analysis was performed by calculating the information entropy during the odor source localization behavior of the silk moth. The link between information entropy and odor source localization was first proposed using infotaxis, which does not rely on odor gradients to locate the odor source (Vergassola et al., 2007). Hernandez et al. (Hernandez-Reyes C. A. et al., 2021) reported the basic concept of using information entropy to analyze insect behavior. In this study, the analytical method of Hernandez et al. was applied to the results of the simultaneous EAG behavior measurement experiments. In this approach, the calculation is based on the timing of the insect’s encounter with the odor rather than on odor concentration. In the EAG data, the timing of the peak (falling timing) of the EAG response was automatically detected using the MATLAB peak detection function (findpeaks, 2023b, MathWorks, MA, United States). The peak was then used as the timing for encountering the odor.

Fundamentally, information entropy $S_{t}$ is calculated from a probability density function, as shown in Equation 1.

$$S_{t}=-\int P_{t}\ln\left(P_{t}\right)dr,$$ (1)

where, r represents the location of the searcher (silk moth). This probability density function is computed based on Bayes’ theorem, as expressed in Equation 2. The likelihood function used in the calculation of the probability density function is updated at each time step based on the encounter rate with the odor, as shown in Equation 3.

$$P_{t}\left(r_{0}\right)=\frac{L\left(\Gamma_{t}|r_{0}\right)}{\int L\left(\Gamma_{t}|r_{x}\right)dr_{x}}$$ (2)

$$L\left(\Gamma_{t}|r_{0}\right)=e^{\left(-\sum \int_{V_{i}}R\left(r_{t^{\prime }}|r_{0}\right)t^{\prime }\right)}\overset{n}{\underset{i=1}{\prod }}R\left(r_{t_{i}}|r_{0}\right)$$ (3)

Here, the $V_{i}$ ’s is the time intervals of absence of detections, $r_{0}$ represents the location of the odor source, and $r_{x}$ represents the location of the insect in the x direction at time t . The expected rate of odor encounter, $R\left(r|r_{0}\right)$ , was calculated using Equation 4 based on an estimation of the plume parameters, such as the wind speed W , the emission rate of the source E and the insect position in x direction.

$$\begin{array}{ccc} R\left(r|r_{0}\right) & = & \frac{aE}{\left|r-r_{0}\right|}e^{\left(\frac{-|r-r_{0}|}{\lambda }\right)}e^{\left(\frac{\left(x-x_{0}\right)W}{2D}\right)}\\ \lambda & = & \sqrt{\frac{D\tau }{1+W^{2}\tau /\left(4D\right)}} \end{array}$$ (4)

**FIGURE 5:**
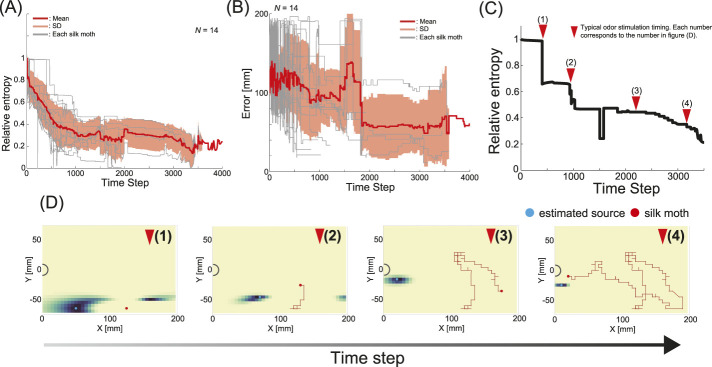
Results of information-theoretic analysis. **(A)** Changes in information entropy across all trials. **(B)** Changes in the error between the true odor source and the estimated source for all individuals. For **(A,B)**, grey represents trials for each individual, the red line represents the mean value, and the light red area represents the standard deviation. **(C)** Changes in information entropy for the results in Figure 3C. **(D)** Changes in the estimated location of the odor source when calculating information entropy. The numbers represent the same as those in **(C)**.

Essentially, the basic principle of infotaxis is that when the searcher is at $P_{t}\left(r_{0}|r\right)$ at time t , the odor source is represented as a probability density function, in which the odor source is located at $r_{0}$ . Herein, the parameters used to calculate the information entropy are as follows: agent sensor size a=10 mm, emission rate E=1 , particle lifetime τ=6.3 , and particle difficulty D=0.057 . These values were obtained from Ref. (Vergassola et al., 2007; Hernandez-Reyes C. A. et al., 2021). Relative entropy is the ratio of the entropy at a certain time step to the entropy at the beginning of the search, i.e., $S_{t}/S_{0}$ . Figure 5A shows the relative entropy trends of all insects. The gray line in Figure 5A represents the relative entropy of each trial, the red line represents the mean, and the thin red area represents the standard deviation. After the search began, we found that the insects all migrated with reduced information entropy. This suggests that silk moths may approach the target odor source by using the timing of the odor encounter as an indicator.

Estimating the probability of the odor source (i.e., the estimated position of the odor source) is possible while calculating the information entropy. Hence, we visualized the positions at which the silk moth estimated the odor source location during localization. Notably, it is unlikely that a similar mapping of the estimated location of the odor source occurs in the brain of the silk moth, which has a smaller number of neurons, and that the visualization of the estimated location in this study was simply introduced as an analytical method. Figure 5B shows a graph showing the time series changes in the error between the true odor source and the estimated source location for 14 silk moths. The gray line in Figure 5B represents the relative entropy of each trial, the red line represents the mean, and the thin red area represents the standard deviation. On average, the error tended to be smaller 5 s before localization. Figure 5C and (D) show the results of calculating the estimated odor source location (see Supplementary Video S2). The time series changes in information entropy in Figure 5C are related to the estimation results in Figure 5D, and the numbers on the triangles in Figure 5C correspond to the results in Figure 5D. Moreover, this trajectory follows the same data as in Figure 3C.

The odor source location was rather diffused in the early stages of localization when no information had been accumulated. However, as localization progressed and more odor information was gathered, the estimated odor source position moved closer to the actual odor source location. Finally, the odor source was estimated to be close to its actual position, suggesting that even the silk moth, which typically reacts reflexively to odor stimuli, possesses a strategy for efficiently approaching the odor source.

Thus, we suggest that selecting behavior appropriately based on the timing of odor encounters rather than simply moving in the direction of the strongest odor is adaptive for effective odor source localization. This study contributes to the understanding of the excellent navigation capabilities of insects by developing an insect-tracking robot system, enabling the measurement of the relationship between odor reception and behavior during the odor source localization of the silk moth, and analyzing the interrelation between them.

## 6 Discussion

All organisms, regardless of their size, possess the navigational abilities required to reach their destinations. This study primarily focuses on tasks involving navigation, where olfaction plays a dominant role. As odors propagate through space via the air, their diffusion is strongly influenced by wind conditions. Consequently, obstacles blocking the wind may lead to entirely different odor behaviors compared with open spaces. Therefore, the device used to track the insect searching for an odor source must be positioned away from the insect to avoid acting as an obstacle. Previous studies proposed an insect-following robot system capable of omnidirectional movement using omni-wheels, which was employed to investigate the phototaxis of pill bugs (Shirai et al., 2022). This robot system has the advantage of being self-propelled, which means it can follow pillbugs infinitely. However, the system is unsuitable when targeting a sensory stimulus, such as an odor, as in this case, because the wheels required for self-propulsion complicate the diffusion of the odor. In this study, we adopted an X–Y stage model that ensures that no obstructions surround the insect, thus providing a setup that closely resembles an open space. This setup offers the advantage of investigating the olfactory capacity of the insect while maintaining a close-to-natural environment. However, the X–Y stage in this study is limited to a size of 1.5×1.5 m, which presents a problem: if the insect moves beyond this area, following it becomes unfeasible. This issue is one of the limitations of our study. Another option, as seen in a previous study (Pannequin et al., 2020), is to robotize the entire laboratory space and continue following the insects. However, such an approach would be costly in terms of space and financial resources, making it impractical. Furthermore, the need to install pillars for cameras that continuously track the insect could create air vortices, potentially disturbing the surrounding environment of the insect. Another limitation of this study is that the moving silk moth and EAG could not be recorded from exactly the same space. As a method for elucidating the relationship between sensory stimuli and behavioral output during odor-source localization, one potential approach involves presenting a light field that simulates odor plumes to optogenetically engineered silk moths, following precedent set by prior studies in *Drosophila* (Kadakia et al., 2022), and subsequently analyzing their localization behavior. This method allows us to confirm how behavior changes depending on the odor flow and strength, but it has the weakness of indirectly measuring odor sensation without looking at the actual physiological response of the antennae. Alternatively, this problem may be solved by using an engineering tool called the insect-controlled robot system that we have proposed (Shigaki and Ando, 2024). The insect-controlled robot is a system that adds mobility to a conventional tethered behavior measurement device; therefore, electrodes can be inserted into the antennae of the insect to stably measure EAG, and the insect can realize odor source localization by controlling the robot. For larger and more robust insects or animals, telemetry could be used to measure neural responses (Ando et al., 2002; Fehlmann and King, 2016). However, developing telemetry for smaller and less powerful insects will be necessary in the future.

An analysis of the relationship between the electroantennogram (EAG) and the behavior measured by our robotic system reveals a tendency for a stronger EAG amplitude during the initial stages of localization rather than near the odor source when the insect is farther from the source. An examination of the EAG response to periodic odor stimuli revealed that silk and hawk moths both exhibit a reduced amplitude (Martinez et al., 2014; Shigaki et al., 2020). As shown in Figure 2E, upon reviewing these time-series data, we found that, while a large amplitude was generated in response to the initial odor stimulus, subsequent stimuli led to a gradual attenuation of the amplitude. It has been reported that within the primary olfactory center, the antennal lobe, neural responses tend to exhibit attenuation following repeated odor stimulations, in comparison to the initial odor stimulus (Fujiwara et al., 2014). This suggests that insect olfaction may exhibit nonlinear characteristics in response to periodic odor stimuli. This nonlinearity could explain why the EAG amplitude was the largest during the initial stages of localization and why it did not reach its maximum even as the insect approached the odor source. Intuitively, in situations where the odor concentration is extremely high near the source, constant sensitivity may cause the insect to lose its directional orientation, making it difficult to discern the correct direction of the odor source. As the male approaches the vicinity of the odor source, it is imperative that he reliably localize the female and initiate mating behavior. Consequently, if the male silk moth continues to move at high speed, it may lose accuracy and the certainty of its localization to the female may decrease. Thus, while a coarse searching strategy may be advantageous in the initial phase, it becomes increasingly important to adopt a more cautious and higher-resolution search as the male nears the target. This modulation of search granularity—from sparse to dense—has also been reported to enhance odor source localization performance in robotic systems (Shigaki et al., 2017), suggesting that it constitutes a critical factor in solving localization tasks. Therefore, it is plausible that this nonlinear characteristic plays a significant role in odor-source localization. Investigating the relationship between the nonlinear characteristics of insect olfaction and their ability to localize odor sources is a key direction for future research. To investigate the nonlinearity at the sensor level, one effective approach would be to use the experimental framework in which an insect antenna is utilized as an odor sensor to control robots or drones (Martinez et al., 2013; Anderson et al., 2020). Moreover, it remains unresolved whether the insect brain indeed modulates its olfactory threshold during odor-source localization because our study assessed only the relationship between EAG responses and behavior. Addressing this question will require future experiments involving electrophysiological recordings or imaging techniques to monitor neural activity within the brain in real time.

Methods for analyzing the behavioral experimental data of organisms, including insects, using an information-theoretic approach have recently become increasingly prevalent (Maekawa et al., 2020). By applying an information-theoretic analysis, it is possible to uncover new insights that may not be revealed through traditional statistical methods. In this study, we quantitatively analyzed the odor-source localization strategy of an insect based on information entropy, revealing that reflexive behaviors are appropriately modulated in response to sensory stimuli, allowing the insect to move toward directions where odor acquisition was likely. In the calculation of information entropy, the present study identified peaks in the EAG responses and utilized these as indicators of odor detection timing. However, depending on environmental factors such as wind dynamics, the frequency of odor release, and the distance from the odor source, the EAG signal may not always exhibit distinct peaks. Given such potential conditions, it seems that insects detect odors by setting a threshold for the intensity of the odor signal they receive. In-depth investigation of the information processing required for odor detection will also contribute greatly to elucidating robust localization behavior. When implementing biological intelligence as an algorithm in the context of robotics, it is essential to understand the criteria by which organisms select their behavior. One approach to achieving this understanding is inverse reinforcement learning (IRL), which has been used to model behaviors such as the migratory patterns of seabirds (Hirakawa et al., 2018), the thermotactic behavior in the nematode *C. elegans* (Yamaguchi et al., 2018), and to estimate reward functions in a moth (Hernandez-Reyes C. et al., 2021). A key advantage of IRL is its ability to derive the reward function governing decision-making when considering an organism as an expert. This enables a generalized representation of the decision-making process. Moving forward, a promising approach would be to analyze and model these behavioral experimental data using machine learning techniques, ultimately aiming to develop more generalized and capable robotic systems.

## Data Availability

The raw data supporting the conclusions of this article will be made available by the authors, without undue reservation.
